# Neurons in auditory cortex integrate information within constrained temporal windows that are invariant to the stimulus context and information rate

**DOI:** 10.1101/2025.02.14.637944

**Published:** 2025-02-14

**Authors:** Magdalena Sabat, Hortense Gouyette, Quentin Gaucher, Mateo López Espejo, Stephen V. David, Sam Norman-Haignere, Yves Boubenec

**Affiliations:** 1 Laboratoire de Neurosciences Cognitives et Computationnelles, Département d’études cognitives, INSERM, Ecole Normale Supérieure, PSL University, Paris, France; 2 Laboratoire des systèmes perceptifs, Département d’études cognitives, École normale supérieure, PSL University, Paris, France; 3 Sorbonne Université, CNRS, Inserm, Institut de la Vision, F-75012 Paris, France; 4 Oregon Hearing Research Center, Department of Otolaryngology-Head & Neck Surgery, Oregon Health & Science University. Portland, USA.; 5 Department of Biostatistics & Computational Biology, Department of Neuroscience, University or Rochester Medical Center, Rochester, USA; 6 Department of Brain & Cognitive Sciences, Department of Biomedical Engineering, University or Rochester, Rochester, USA

## Abstract

Much remains unknown about the computations that allow animals to flexibly integrate across multiple timescales in natural sounds. One key question is whether multiscale integration is accomplished by diverse populations of neurons, each of which integrates information within a constrained temporal window, or whether individual units effectively integrate across many different temporal scales depending on the information rate. Here, we show that responses from neurons throughout the ferret auditory cortex are nearly completely unaffected by sounds falling beyond a time-limited “integration window”. This window varies substantially across cells within the auditory cortex (~15 to ~150 ms), increasing substantially from primary to non-primary auditory cortex across all cortical layers, but is unaffected by the information rate of sound. These results indicate that multiscale computation is predominantly accomplished by diverse and hierarchically organized neural populations, each of which integrates information within a highly constrained temporal window.

## Introduction

Temporal integration is central to nearly all aspects of auditory perception. A key challenge is that information in natural sounds is distributed across a vast range of timescales. Speech and animal vocalizations, for example, are characterized by time-varying changes in pitch and spectrotemporal structure that unfold over tens of milliseconds, which are then assembled into longer sequences across hundreds of milliseconds or seconds (Kello et al., 2017). Importantly, the rate at which this information varies is itself highly dynamic (Pellegrino et al., 2011). The rate of spectrotemporal changes, for example, is constantly varying, even within a vocalization, and the duration and density of vocalizations that compose a sequence is also highly variable. Thus, to derive information from complex sounds such as speech and animal vocalizations, the auditory system must be able to flexibly integrate across multiple timescales.

One hypothesis is that neurons in the auditory cortex can flexibly vary the time window over which they integrate information, depending on the rate at which information is varying. This hypothesis is motivated, in part, by prior work showing neurons in the auditory cortex perform flexible, context-dependent computations (J. Fritz et al., 2005; J. B. Fritz et al., 2007; Spitmaan et al., 2020). Artificial neural networks increasingly rely on flexible and nonlinear computational units whose temporal integration window can dynamically vary (Keshishian et al., 2020; Keshishian & Norman-Haignere, 2021), and such systems have shown promise as models of neural computation in the brain (Cichy & Kaiser, 2019; Esposito et al., 2024; Kell et al., 2018; Kietzmann et al., 2019; Li et al., 2023; Yamins & DiCarlo, 2016). A second hypothesis is that individual neurons integrate across largely fixed timescales, but instantiate a diverse array of these integration scales across cells so as to support flexible multiscale integration across the neural population (Perez-Nieves et al., 2021). This hypothesis is motivated in part by prior work suggesting that cortical neurons show a diverse range of temporal scales even within a single cortical region (Cavanagh et al., 2020; Elgueda et al., 2019; T. Lu et al., 2001; Ulanovsky, 2004).

Distinguishing between these hypotheses has been challenging, in part due to the difficulty of measuring integration windows from highly nonlinear systems such as the brain. Integration windows are often defined as the time window analyzed by a sensory system, and thus the window within which stimuli can alter the neural response (Chen et al., 2012; Lerner et al., 2011; Poeppel, 2003; Theunissen & Miller, 1995). Although this definition is simple and general, there have been no simple and general methods for estimating integration windows. Neurons in the auditory system are often modeled using spectrotemporal receptive fields (STRFs), which assume a fixed, linear mapping between a spectrogram and the neural response. STRFs, however, cannot model dynamic nonlinear processing, which is prominent in the cortex (Harper et al., 2016; Meyer et al., 2016; Mizrahi et al., 2014; S. V. Norman-Haignere & McDermott, 2018), and likely plays an important role in flexible, multiscale integration (Deneux et al., 2016; Dubreuil et al., 2022; Kell et al., 2018; Keshishian et al., 2020).

**Figure 1 |:**
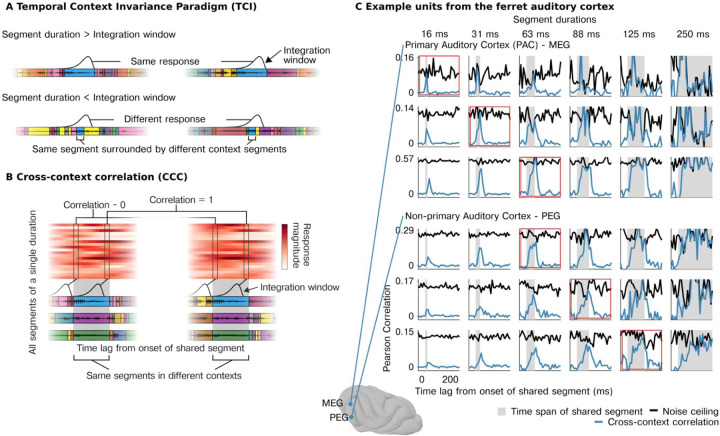
Neurons in auditory cortex show context-invariant responses. **A,** Segments of natural sounds were presented in different orders, leading to the same segment (blue) being surrounded by different “context segments”. **Top row**, If the neural response has an integration window that is less than the segment duration, there will be a moment when the window is fully contained within the shared segment yielding the same response across both contexts. Waveforms for different segments are schematically indicated by colored boxes and the integration window is plotted at the moment of maximum overlap between the two contexts. **Bottom row**, If the shared segments are shorter than the integration window, then the surrounding context segments can alter the response. **B**, Schematic of the “cross-context correlation” (CCC) analysis used to estimate context invariance. Response timecourses surrounding each segment are organized as a segment-by-time matrix (aligned to segment onset), separately for each context. We then correlate responses across segments between the two contexts for a given lag, which is equivalent to correlating corresponding columns (connected boxes) across matrices from different contexts (see text for details). A noise ceiling for the CCC is computed by performing the same analysis when the context is identical (using repeated presentations of each sequence). **C**, Example of the CCC (blue) and noise ceiling (black) from example multi-units in primary (MEG, top) and non-primary (PEG, bottom) ferret auditory cortex. For all units, there is a lag and segment duration for which the CCC equals the noise ceiling, indicating a context-invariant response. The segment duration needed to achieve a context-invariant response varies substantially across units.

In this study, we leveraged a recently developed paradigm for measuring integration windows from nonlinear (or linear) systems (the temporal context invariance or TCI paradigm) (Figure 1A). The paradigm directly tests the definition of an integration window by determining whether there is a stimulus duration for which the response is invariant to surrounding context stimuli. As a consequence, the method does not make any assumptions about the features that underlie the response (e.g., a spectrogram) or the relationship between those features and the neural response (e.g., linear or nonlinear). The TCI paradigm was recently validated using artificial deep neural networks (Keshishian & Norman-Haignere, 2021) and macroelectrode recordings from the human auditory cortex (S. V. Norman-Haignere et al., 2022). Macroelectrodes, however, average activity across thousands of neurons within a local area (Baratham et al., 2022; Leonard et al., 2024; Manning et al., 2009; Ray & Maunsell, 2011), which makes it impossible to determine if multiscale integration is accomplished by diverse populations of neurons integrating across largely fixed timescales, or flexible integration within individual neurons.

In this study, we applied the TCI paradigm to microelectrode recordings from the ferret auditory cortex using ferret vocalizations, speech, and music across three experiments. We first tested whether cells in the auditory cortex showed evidence of a well-defined integration window, beyond which stimuli have little effect on the response, and if so, how this window varies across the neural population. We then examined whether integration windows reflect absolute time or vary with the information rate of sound by systematically manipulating the information rate using stretched and compressed sounds.

## Results

### Neurons in the auditory cortex integrate information within constrained temporal windows

We characterized integration timescales of neurons in the auditory cortex of awake ferrets. We presented sequences of sound segments in different orders, such that each segment was surrounded by distinct “context” segments (Figure 1A). We note that while “context” has many meanings (Angeloni & Geffen, 2018), we operationally define context in our paradigm as the stimuli that surround a segment. We test the hypothesis that there is a minimum time window beyond which surrounding stimuli no longer alter the response. If a neuron has a well-defined integration window that is less than the segment duration, there will be a moment when the window is fully contained within the shared segment and thus by definition unaffected by the surrounding segments.

We measured context invariance using the “cross-context correlation” (CCC). For a given segment duration, we aligned the response timecourses surrounding each segment in a segment-by-time matrix, separately for each of two contexts (Figure 1B). We then correlated corresponding columns across matrices from differing contexts (CCC, connected boxes in Figure 1B). For a given time lag relative to segment onset, the CCC measures the similarity of the response pattern across segments between the two contexts. At segment onset, the integration window will overlap the preceding segments, which are random across contexts, and the CCC should be near 0. If the integration window is less than the segment duration, there will be a time lag when the window is fully contained within the shared segment, leading to a correlation of 1 for a noise-free response. To account for noise, we measured a ceiling for the CCC by computing the same correlation when the context was identical using repeated presentations of the same sequence. Longer integration windows will cause the peak of the CCC to drop relative to the noise ceiling and will also cause a more gradual build-up and fall-off since it takes longer for the integration window to enter and exit the shared segment.

We plot the CCC and noise ceiling for multi-units from different regions of the ferret auditory cortex (Figure 1C; see Supplementary Figure 1 for additional units). Strikingly, we found that for virtually all cells with a reliable response to sound, there was a segment duration and lag for which the CCC reached the noise ceiling, indicating a nearly fully context-invariant response. Once the CCC reached the noise ceiling, it tightly tracked the noise ceiling until segment offset, consistent with a temporally compact window composed of a single mode (i.e., not multimodal). These results indicate that nearly all sound-responsive cells in the auditory cortex show a temporally compact integration window beyond which stimuli have little effect on the neural response. Notably, the segment duration and lag needed to achieve a context-invariant response differed substantially across units and cortical fields (Figure 1C). For some units, segment durations as short as 15 ms were sufficient to yield a context-invariant response, while other units required longer segment durations of 32, 64, or 128 ms, particularly in non-primary regions of the auditory cortex (PEG).

### Neural integration windows vary widely across the neural population, increasing in non-primary regions across all cortical layers.

To quantify these effects, we used a previously developed and validated computational model to estimate the integration window of each identified unit (345 units with reliable response to sounds across 3 animals, test-retest correlation r> 0.1; p<10^-5^ via permutation). The model finds a parametric window that best predicts the CCC across all segment durations and lags. The main text figures plot the estimated width of the model window (the smallest interval that contained 75% of the mass). The model also computes an estimated integration “center” (median of the parametric window), which can be thought of as the overall delay between the stimulus and the integration window. The center of the window was highly correlated with the window’s width and close to the minimal possible center given the window width, suggesting that neurons integrate information about as quickly as possible given the time window being analyzed (Supplementary Figure 2).

**Figure 2 |:**
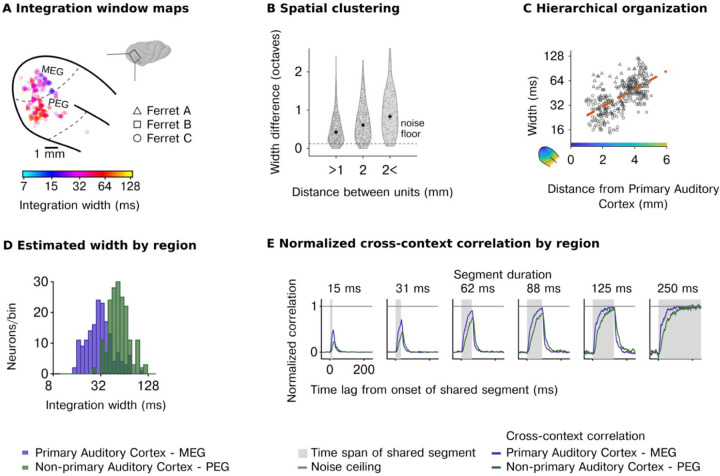
Diverse, hierarchically organized temporal integration windows across the neural population. **A**, Anatomical map of model-estimated integration windows in three animals (window width: smallest interval containing 75% of the window’s mass). **B**, The difference in integration windows between pairs of units as a function of their spatial distance, demonstrating that nearby units have more similar windows. **C**, Integration windows as a function of distance to the primary auditory cortex (PAC) (see color map in inset). **D,** Histograms of integration windows for primary (MEG) and non-primary auditory cortex (PEG) showing substantial diversity across units and hierarchical organization. **E,** Median noise corrected cross-context correlation for all neurons in primary (MEG) and non-primary (PEG) auditory cortex.

This analysis revealed a wide range of integration windows across cells spanning approximately 3 octaves (Figure 2A–D; Supplementary Figure 3 shows analogous results for integration centers). These integration times showed clear anatomical organization (Figure 2A): cells that were anatomically closer showed more similar windows (Figure 2B), and cells further from primary auditory cortex (PAC) showed longer windows (Figure 2C) (${\text{F}}_{1.343}=84.79$, p<0.001, $\beta_{\text{distance}\;\text{to}\;\text{PAC}}=0.35$ octaves/mm, CI = [0.275, 0.424]; N=345), leading to a substantial and significant difference between primary (MEG) and nonprimary (PEG) auditory cortex (Figure 2D) (${\text{F}}_{1.343}=109.06$, p<0.001, $\beta_{\text{region}}=-0.799$ octaves, CI = [−0.949, −0.648]; N=345).

To complement our model-based analyses, we also measured the average CCC in primary and non-primary auditory cortex and normalized this metric by the average noise ceiling, also computed separately for each region (Figure 2E). This analysis replicated our model-based results, with non-primary regions requiring substantially longer segment durations for the CCC to reach the noise ceiling. This analysis further demonstrated that even in non-primary regions, there is a time-limited window (~150 ms), outside of which stimuli have very little effect on the neural response, suggesting that there is a compact integration window that strongly constrains the computations of virtually all neurons in the auditory cortex. These results held across all sound categories tested (Supplementary Figure 4).

**Figure 3 |:**
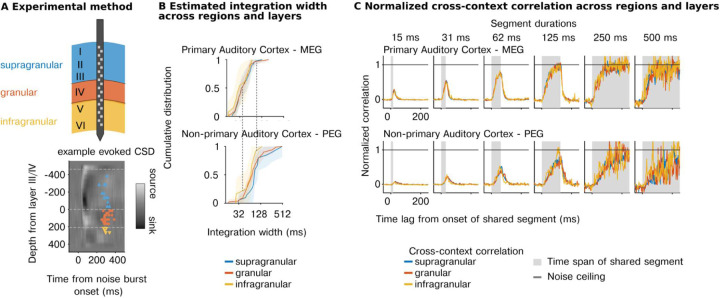
Integration windows show hierarchical organization across regions but not layers. **A,** *Top:* Schematic illustration of recordings from a laminar probe sampling supragranular (blue), granular (red), or infragranular (yellow). *Bottom:* Current Source Density (CSD) plot, evoked with a burst of noise for an example session. Individual markers correspond to recorded units and indicate approximate locations along the laminar probe. The color corresponds to the laminar classification of each unit. The colorbar reflects the spatial distribution of current flow in extracellular space used to estimate the sources and sinks of change in measured potential along the laminar probe. Details of the laminar assignment are described in the method section. **B**, Cumulative distribution of estimated integration windows for primary (top) and non-primary (bottom) regions broken down by layer class (blue: supragranular, red: granular, yellow: infragranular). Dashed lines show corresponding points between plots from different regions so they can be visually compared. Integration windows are longer in non-primary regions but do not differ substantially between layers. **C,** Median noise corrected cross-context correlation separately for each layer (supragranular, granular, infragranular) and region (primary - MEG, nonprimary - PEG).

Our first experiment shows that temporal integration is organized hierarchically across regions, but did not enable us to investigate hierarchical organization between different cortical layers. To address this question, we repeated our experiments using laminar probes (231 cells with reliable response to sounds across 5 animals, test-retest correlation r> 0.1; *p*<10^-5^ via permutation) and grouped cells based on both their region and cortical layer (supragranular, granular, infragranular) using source density analysis (Figure 3A). We replicated our prior findings, showing a substantial increase in integration windows between primary (MEG) and non-primary regions (PEG) (${\text{F}}_{1,21.47}=19.76$, p<0.001, $\beta_{\text{region}}=-0.88$ octaves, CI = [−1.270, −0.490]; N=231, Figure 3B). There was also a significant effect of layer (width: ${\text{F}}_{2,218.86}=6.87$, p=0.001) driven by shorter integration windows in the infragranular layers ($\beta_{\text{infragranular}}=-0.486$ octaves, CI = [−0.796, −0.175]). However, the magnitude of the effect was small both in absolute terms $\left(\Delta M_{\text{width}}=-4.73\;\text{ms}\right)$ and compared with the effect of region (${\text{F}}_{2,37.48}=15.44$, p<0.001). Consistent with these results, the average cross-context correlation was similar between layers but differed across regions with non-primary areas requiring a longer segment duration and lags to reach the noise ceiling (Figure 3C). Thus, the dominant form of hierarchical anatomical organization appears to be region-based and not layer-driven.

### Integration windows reflect absolute time and do not vary with the information rate of sound.

**Figure 4 |:**
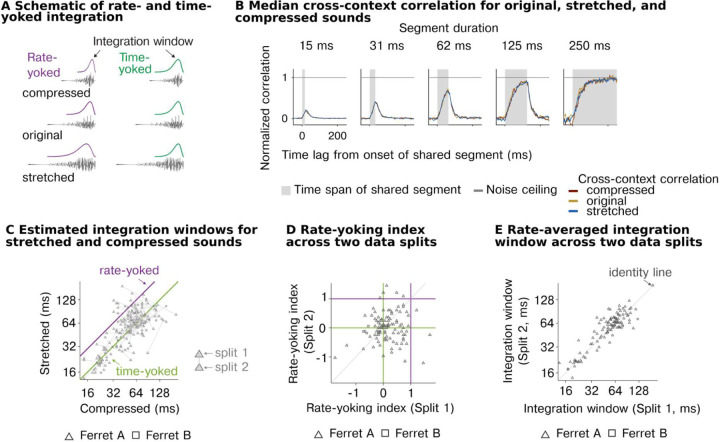
Temporal integration is invariant to information rate. **A**, Schematic illustration of the predictions for time- vs. rate-yoked integration. Stretching and compression will expand and shrink the integration window if it is yoked to the information rate of sound (purple, left) but not if it reflects absolute time (green, right). **B**, Median normalized CCC across all recorded units for stretched, original, and compressed stimuli. **C**, Integration windows for all units for compressed (x-axis) and stretched (y-axis) stimuli. Green and purple lines show the prediction from a time-yoked and rate-yoked response. **D,** Reliability of rate-yoking index across two independent data splits. **E,** Reliability of integration windows averaged across stimuli rates for comparison.

Our analyses so far suggest that neurons in the auditory cortex integrate information within a highly constrained temporal window beyond which stimuli have little effect on the cortical response and that this window varies substantially between neurons across the population. We next tested whether these integration windows reflect absolute time or instead vary with the information rate of the sound. To answer this question, we systematically altered the duration and rate at which sound information varies by stretching and compressing sounds by a factor of $\sqrt{2}$ (preserving frequency/pitch) (Figure 4A). If the neural integration window varies with information rate (rate-yoked integration), the window should appear to scale with the magnitude of stretching and compression. In contrast, if the integration window reflects absolute time (time-yoked integration), the window will be unchanged by this manipulation. We tested both speech and ferret vocalization because they contain many different forms of time-varying structure that are ecologically relevant to the animal (particularly for ferret vocalizations). We focused our experiments on a non-primary region (PEG) because we expected rate-yoked integration, if present, to be strongest in higher-order regions (Atiani et al., 2014; Elgueda et al., 2019).

As a simple summary metric, we first measured the average cross-context correlation for all units from non-primary auditory cortex for each of the different rates, pooling data from both sound categories (Figure 4B). We found that the average CCC was virtually identical across the three rates for all segment durations tested, suggesting integration windows reflect absolute time and do not vary with the information rate. We next quantitatively compared the integration windows for different stimulus rates, separately estimated for each cell using our computational model (Figure 4C). If the integration windows are fully rate-yoked, integration windows for stretched sounds should be twice as long on average as the integration windows for compressed sounds, yielding an octave shift on a logarithmic scale (Figure 4C, purple line). If the integration windows are time-yoked, the windows should cluster around the line of unity (Figure 4C, green line). We found that the estimated windows clearly clustered around the line of unity, consistent with our previous analysis suggesting time-yoked integration dominates. These effects were reliable across both of the two sound categories tested (Supplementary Figure 5) and across all pairs of sound rates (e.g., original vs. compressed, original vs. stretched).

There was substantial variation from cell to cell in the estimated integration window for stretched and compressed speech (Figure 4A). To test if this variation reflected genuine variation in rate yoking or just variation due to unreliable sources of noise, we measured the integration window for each cell and rate using two independent splits of data. We then computed a measure of rate yoking separately for each data split, and correlated this measure across units between the two splits (Figure 4D). Rate yoking was measured by subtracting the integration window for stretched and compressed speech on a logarithmic scale and dividing by the 2-octave difference in rates. The metric thus provides a simple, graded measure of the extent of time-yoked (0) vs. rate-yoked integration (1) (note that due to noise the metric can exceed the 0 and 1 bounds). The effect of rate of the stimuli was marginally significant (width: ${\text{F}}_{2,664.58}=2.53$, p=0.08, $\beta_{\text{strerched}}=0.16$ octaves, CI = [0.019, 0.308], $\beta_{\text{compressed}}=0.105$ octaves, CI = [−0.039, 0.249]; N = 702 (observations for 117 neurons)), however, we found that the variation from cell to cell in the strength of rate yoking was not reliable across data splits (Spearman rank correlation of 0.09, p = 0.52 via bootstrapping), indicating that it mostly reflected unreliable source of noise in the data (Figure 4D). In contrast, the overall integration window, averaged across rates, was highly reliable (Spearman rank correlation of 0.72, p < 0.0001 via bootstrapping) (Figure 4E). These results demonstrate that neural integration windows do not vary with the information rate of sound, and thus that multiscale integration is accomplished by diverse populations of neurons each of which integrate information within a highly constrained temporal window.

## Discussion

Our study demonstrates that neurons in the auditory cortex integrate information across a time-limited window beyond which sounds have very little influence on the cortical response. This window varies by approximately 3 octaves (~15 to 150 ms) across cells in the auditory cortex and exhibits clear anatomical organization, with two to three times longer integration windows in non-primary regions and little difference within a cortical column. In contrast, these windows do not vary with the information rate and duration of sound structures (e.g., vocalizations), suggesting that they are a fundamental property of the auditory system and not a property of the sounds analyzed by the auditory system. These results indicate that multiscale computation is accomplished by diverse, hierarchically organized populations of neurons, each of which integrates information across a specific, time-limited, and context-invariant temporal window.

### Implications for models of multiscale computation.

Understanding the computations that enable the auditory system to integrate across the multiscale temporal structure of natural sounds is an important goal of auditory neuroscience. Multiscale integration is computationally challenging in part because information in sound is organized across many different timescales, and these timescales are highly variable and context-dependent.

Our results show that virtually all neurons in the auditory cortex integrate information with a compact, temporal window beyond which stimuli have very little effect on the neural response. This finding is highly nontrivial, since highly nonlinear, recurrent systems such as the brain are capable of integrating across potentially very long timescales (Keshishian & Norman-Haignere, 2021; Mante et al., 2013). Our results suggest that this window is temporally compact, since multi-peaked windows would produce oscillations in the cross-context correlation, which were not observed empirically. This window is also about as fast as it can be with window centers close to the minimum possible for a causal system. These results suggest that temporal integration windows strongly constrain and shape the computations performed by the auditory cortex.

Our second key result is that integration windows show substantial heterogeneity across the neural population, with nearly 3 octaves of variation. We show that this variation is hierarchically organized across regions, but not across cortical layers. We note that increasing integration windows are not an inevitable consequence of hierarchical organization (as evidenced by the weak effect of layer). For example, hierarchical recurrent neural networks can show similar integration windows across their layers (Keshishian & Norman-Haignere, 2021), and training RNNs on challenging auditory tasks does not necessarily induce increasing integration windows across layers (S. Norman-Haignere et al., 2024). We note that while we observe a substantial increase in integration windows between regions there is still substantial heterogeneity in integration windows within a region that exhibits anatomical clustering, suggesting that within a region the auditory cortex performs multiscale temporal analysis using spatially organized neural populations.

Critically, we provide evidence that the temporal windows of neurons in the auditory cortex do not vary with the rate at which information is varying in natural sounds. In particular, we find that temporal windows on average are completely unaffected by stretching and compression, even though stretching and compression changes the rate at which virtually all acoustic structure varies, as well as the duration of all sound events (e.g., vocalizations). This result is also highly nontrivial. Nonlinear systems are highly capable of flexibly varying their integration window and our TCI method is sensitive to these types of changes and can robustly detect rate-yoked integration when present (Keshishian & Norman-Haignere, 2021). Collectively, these results demonstrate that multiscale computation is performed by diverse populations of neurons, each of which integrate information within a highly constrained temporal window beyond which stimuli have very little effect on the neural response and which is invariant to the information. These results suggest that flexible temporal integration is accomplished by flexibility at the neural population level rather than flexibility within single neurons.

Our finding of hierarchical organization is consistent with a prior study that also observed hierarchical organization in human auditory cortex (S. V. Norman-Haignere et al., 2022). This prior study used macroelectrodes which pool activity from thousands of neurons and thus was not able to test whether individual neurons show a time-limited integration window, characterize the heterogeneity across the neural population, or examine layer-wise effects. Prior work has found that the functional organization of the human auditory cortex differs substantially from that of ferrets (Landemard et al., 2020), likely in part due to the unique importance of speech and music to human hearing, and thus a priori, it was entirely possible that we might have observed a very different organization in ferrets. Our findings therefore provide novel evidence that region-based hierarchical integration, in which longer timescale computations are performed using the output of shorter timescale computations, is a fundamental property of biological auditory systems that is not limited to the human auditory cortex.

The integration windows observed in this study are shorter than those observed in the human auditory cortex, which range from about 50 to 400 ms. This difference could reflect differences in the neural measures employed (e.g., macro- vs. micro-electrodes) or could reflect species differences. For example, prior work shows that neural populations with longer integration windows (< 200 ms) in the human auditory cortex show strong selectivity for speech and music ((S. V. Norman-Haignere et al., 2022)), which is absent from ferret auditory cortex (Landemard et al., 2020), suggesting that these longer timescale may have evolved or developed to process speech- or music-specific structure.

### Relationships to other types of neural timescales.

One approach to characterizing the sensory responses is to attempt to learn an explicit functional mapping (or “encoding model”) between the stimulus and the neural response. Neural responses in the auditory system have been classically modeled using spectrotemporal receptive fields which fit a linear mapping between a time-frequency spectrogram-like representation and the neural response (Calabrese et al., 2011). STRFs, however, are unable to predict much of the neural response variance in the auditory cortex, particularly for natural sounds, due to prominent nonlinearities between the spectrogram and the neural response (Sadagopan & Wang, 2009) that are not captured by the model (David et al., 2009; Keshishian et al., 2020). Some prior studies have fit more complex encoding models, such as deep neural networks (Harper et al., 2016; Keshishian et al., 2020), but these models still fail to explain much of the neural response, and it is not clear how to measure integration windows from these types of complex, nonlinear models. In contrast, our paradigm does not make any assumptions about the features that underlie the response (e.g., spectrogram) or how these features are mapped to the neural response, and thus is able to characterize the integration windows from highly nonlinear systems such as deep artificial neural networks (Keshishian & Norman-Haignere, 2021).

An alternative approach that does not require fitting a complete encoding model is to simply measure temporal characteristics of the neural response to a sound. For example, prior studies have measured the rate of temporal modulations in the neural response (Bartlett & Wang, 2007), which has revealed that the upper frequency limit at which the neural response will “phase-lock” to a stimulus frequency slows from the periphery to the cortex. Phase locking, however, does not provide a measure of the neural integration window since for example a fast neural modulation could be produced integrating information across both a long or short window (e.g., integrating across many cycles of an oscillation or just a single cycle). Other studies have attempted to measure the time period in the neural response that contains information about a stimulus or stimulus property (sometimes referred to as the “encoding window”) (Theunissen & Miller, 1995). While useful, this measure is highly stimulus dependent and will vary with the duration and information rate of sounds. Other studies have measured the extent to which the neural response timecourse expands or contracts as the stimulus is stretched or compressed (Lerner et al., 2014). However, the neural response will tend to expand and contract with the stimulus even if the integration window remains fixed and this metric thus does not provide a good estimate of the integration window or whether this window varies with the information rate. By contrast, the TCI method can directly estimate the neural integration window, independent of many stimulus properties such as the information rate and duration of sounds, as we show here.

Recent research has characterized the “intrinsic timescales” of neural systems, often by measuring the autocorrelation of the neural response after removing stimulus-driven changes (Chaudhuri et al., 2014; Fontanier et al., 2022; Manea et al., 2022; Murray et al., 2014; Zeraati et al., 2023). Intrinsic timescales are distinct from integration windows, since they do not directly specify anything about the relationship between the stimulus and the neural response. In contrast, integration windows are fundamentally defined with respect to the stimulus. Empirically, however, intrinsic timescales have been shown to increase across the cortical hierarchy, potentially analogous to the hierarchical organization that we observe here. Thus, one possibility is that there are shared underlying neurobiological mechanisms (e.g., changes in synaptic integration time constants) that couple intrinsic timescales and stimulus integration windows, and cause both to increase across the cortical hierarchy. Future research could explore this link by applying the methods used here to computational models of intrinsic timescales (i.e. (Zeraati et al., 2022)), which is feasible because our method is effective in nonlinear models.

### Methodological choices, limitations, and future directions.

Many prior studies have demonstrated stimulus-driven effects that extend beyond the integration timescales observed in this study. For example, stimulus-specific adaptation effects have been observed over multi-second timescales (Herrmann et al., 2015; Ulanovsky, 2004; Ulanovsky et al., 2003; Valerio et al., 2024), and effects of learning and memory can be observed across minutes, hours, and days (Elgueda et al., 2019; K. Lu et al., 2018). Thus, there are likely multiple timescales over which stimuli can influence the neural response, in part reflecting different neurobiological mechanisms (e.g., adaptation, learning, memory, etc.). Our results demonstrate that the cumulative effects of these longer timescale processes are small during the processing of natural sounds compared with the effects of stimulus variation within the window. Indeed, we find that for virtually every unit in the auditory cortex there is a time period from approximately 15 to 150 milliseconds, where the cross-context correlation closely tracks the noise ceiling, indicating that surrounding stimuli have virtually no effect on the neural response. One caveat is that the noise ceiling measured by the TCI paradigm will reflect all responses that are reliable across a long sequence of sounds. Thus, any neural responses that change between two presentations of the same sequence will be treated as noise. As a consequence, if there were learning and memory processes that took place across repetitions of an entire sequence (Bartlett & Wang, 2005) our paradigm would not be sensitive to them.

We used uniform stretching and compression (preserving pitch) because this alters the information rate and duration of virtually all sound properties by a known magnitude, which is useful because we do not know which features are important to the neural response. Recently, we showed that this approach is able to reveal a near complete transition from time-yoked to rate-yoked information across the layers of highly nonlinear deep artificial neural network (DANN) trained on challenging speech recognition tasks, even though the models were only ever exposed to natural speech without any compression or stretching. This finding demonstrates that our method can cleanly detect rate-yoked integration, even from a highly complex nonlinear system, where the features and structures that underlie the system’s response are unknown. Nonetheless, a potential limitation of our approach is that uniform stretching and compression are not entirely natural, and it is possible that biological systems might show greater evidence of rate-yoked computation for more natural rate manipulations.

Our study focused on characterizing integration windows in the auditory cortex, across multiple regions and layers. An important question for future research is how integration windows are structured beyond the auditory cortex in higher-order cognitive and motor regions, as well as in associative multisensory regions. It is possible that stimulus-driven integration windows continue to grow beyond the auditory cortex (Chaudhuri et al., 2015; Manea et al., 2022; Murray et al., 2014), but remain yoked to absolute time. Another possibility is that integration windows become increasingly flexible outside of the auditory cortex in order to support adaptive computations that underlie perception and behavior (Piet et al., 2018). These types of questions could be answered by applying our paradigm to neural responses beyond the auditory cortex, testing longer segment durations and using multimodal stimuli.

## Methods

The data from the paper come from three experiments. Experiment I compared integration windows between different regions using natural speech, music, and ferret vocalizations. Experiment II examined layer effects. Experiment III examined the importance of the information rate by comparing original, stretched, and compressed sound stimuli. Experiments I and III were conducted at the École Normale Supérieure (ENS) using chronic multi-electrode arrays, and Experiment II was conducted at Oregon Health & Sciences University (OHSU) using laminar probes. The neurophysiological recording methods for chronic arrays and laminar probes are described separately.

### Neurophysiological recordings with chronic arrays at ENS

#### Animal preparation.

Experiments were performed in adult female ferrets (Mustela putorius furo, ferret A, B, and C) across one or both hemispheres (ferret A: left and right, ferret B, right, and ferret C: left). The animals were 1–3 years of age, weighing 500–1000 g, and were housed in pairs or trios with a normal day-night light cycle and unrestricted access to food and water. All ferrets were virgins. Experiments were approved by the French Ministry of Agriculture (protocol authorization: 21022) and all manipulations strictly comply with the European directives on the protection of animals used for scientific purposes (2010/63/EU).

#### Surgical procedure.

Chronic neurophysiological recordings were performed head-fixed after implantation of a metal headpost attached to the skull (detailed procedure in (Chillale et al., 2023)). After recovery from the initial headpost surgery, chronic floating multielectrode arrays were implanted in a subsequent surgery. A 10 mm x 10 mm craniotomy was performed under anesthesia (1% isoflurane) over auditory cortex. The large craniotomy allowed us to identify the different regions of the auditory cortex (anterior/middle/posterior ectosylvian gyri) by visual inspection of the pseudosylvian and suprasylvian sulci and additional landmarks. The animals were then implanted with floating multielectrode arrays (Platinum/lridium, MicroProbes, electrodes of impedance of 500 or 750 kΩ, 4 rows of 8 electrodes with 0.4 μm distance between the electrodes) in primary or non-primary regions using a micromanipulator and sealed with Vetbond (3M). When possible the dura was pulled over the implanted array. Finally the removed skull flap was put back and sealed with Kwik-Sil (WPI) and bone cement (Palacos, Heraeus). Animals could then recover for 1 week, with unrestricted access to food, water, and environmental enrichment.

#### Recording procedure.

Recordings were performed head-fixed in a soundproof chamber. Continuous electrophysiological recordings were digitized, amplified, and recorded at 30 kHz using a digital acquisition system (OpenEphys). We presented sounds to the animals via custom scripts implemented using a MATLAB toolbox designed for low-latency sound delivery (PsychToolbox). We collected 19 recording sessions in Ferret A, 45 in ferret B, and 40 in ferret C.

#### Preprocessing.

Electrode responses were common-averaged to the grand mean across all functional electrodes of each array. We applied a 300–6000 Hz band-pass filter (2^nd^ order Butterworth filter with 3dB cutoff applied forward and backward). We then detected multi-unit activity by thresholding the signal (3 SD) and removing artifacts using PCA--based customized spike sorting routines written in MATLAB (J. Fritz et al., 2003).

### Neurophysiological recordings with acute laminar probes at OHSU

#### Animal preparation and surgical procedure.

Adult male ferrets (aged 6–9 months) were surgically implanted with a head post to stabilize the head and enable multiple small craniotomies for acute electrophysiological recordings. Anesthesia was induced with ketamine (35 mg/kg) and xylazine (5 mg/kg) and maintained with isoflurane (0.5–2%) during the surgical procedure. The skin and muscle on top of the cranium were removed and the surface of the skull was cleaned. Ten to twelve small surgical screws were placed on the edge of the exposed skull as anchor points. The surface of the skull was chemically etched (Optibond Universal, Kerr) and a thin layer of UV-cured dental cement (Charisma Classic, Kultzer) was applied over the exposed surface. Two stainless steel head posts were aligned along the midline and embedded with additional cement. Finally, cement was used to build a rim extending out from the edges of the implant. The rim served the dual purpose of holding bandages over the implant margin wounds and creating wells to hold saline over the recording sites. Once the implant was finished, excess skin around it was removed, the wound around the implant was closed with sutures and the animal was bandaged. Antibiotics and analgesics were administered as part of the post-op recovery.

After at least 2 weeks following surgery, the animals were acclimated to a head-fixed posture, during intervals starting at 5 min and increased 5–10 min every day. Food and liquid rewards were given during these acclimation sessions to help the animals relax under restraint. Animals were considered ready for recording when they could be restrained for more than 3 h without signs of distress (e.g., the animals being relaxed enough to fall asleep).

#### Preprocessing.

The putative location of A1 and dPEG was determined during the headpost implantation surgery based on external landmarks: the posterior and medial edges of A1 falling, respectively, 13 mm anterior to the occipital crest and 8 mm lateral to the center line, and dPEG immediately antero-lateral to A1 (J. Bizley et al., 2005). To functionally confirm recording locations, we opened small craniotomies <1 mm diameter and performed preliminary mapping with tungsten electrodes (FH–Co. Electrodes, AM Systems Amp, MANTA software (Englitz et al., 2013)). We measured the tuning of the recording regions using rapid sequences of 100ms pure tones and used tonotopy to identify cortical fields. We specifically looked for the frequency tuning inversion: high-low-high moving in an antero-lateral direction, which marks the boundary between primary (A1) and secondary (dPEG) fields. At tonotopically mapped sites, we performed acute recordings with 64-channel integrated UCLA probes (Du et al., 2011), digital head-stages (RHD 128-Channel, Intan technologies) and OpenEphys data acquisition boxes and software (Siegle et al., 2017). The probes were inserted approximately normal to the cortical surface, up to a depth of ~1 mm from the dura surface. Due to spatial constraints of the recording site and apparatus, penetrations deviated from normality up to 20°. The depth of recording sites and their location in superficial areas A1 or PEG were confirmed by current source density analysis.

Raw voltage traces were processed with Kilosort 2 (Stringer et al., 2019), clustering and assigning spikes to putative single neurons. The clusters were manually curated with Phy (Rossant et al., 2016). Units were only kept for analysis if they maintained isolation and a stable firing rate over the course of the experiment. Unit isolation was quantified as the percent overlap of the spike waveform distribution with neighboring units and baseline activity. Isolation >95% was considered a single unit and kept for analysis. We further filtered neurons based on the reliability of their responses, requiring a Pearson’s correlation >0.1 between PSTH responses to the same stimuli (3–10 repetitions, 20 Hz sampling) drawn from random halves of repeated trials.

### Experimental protocols

#### Experiment I.

The goal of this experiment was to measure integration windows using a diverse set of natural sounds. The stimuli tested included sequences composed of segments of varying durations (ranging from 15.625 to 500 ms). Segments were excerpted from 24 natural sounds (each 500 ms) spanning three different categories (8 sounds/category): speech, ferret vocalizations, and music. Each sound was subdivided to create segments of the desired duration. Each natural sound was RMS-normalized prior to segmentation. We presented the segments in two pseudorandom sequences, with all segment durations and categories interleaved. Sequences varied in duration from 2 to 6 minutes depending on the session and animal. Segments were concatenated using cross-fading to avoid click artifacts (15.625 ms raised cosine window). Each stimulus was repeated several times to measure a reliable noise ceiling (8 repetitions for most recording sessions).

#### Experiment II.

The goal of this experiment was to replicate key findings from Experiment I as well as characterize the organization of integration windows across cortical layers using acute laminar probes. The stimuli tested were similar to those in Experiment I. The segments were excerpted from 20 natural sound recordings that included ferret vocalizations, speech, music, and other animal calls/vocalizations and environmental sounds. The segment durations spanned 16 to 500 milliseconds. The segments for each duration were presented in a separate sequence, using two different random orders. The frequency of the sounds was adjusted so that the mean excitation pattern across sounds was approximately white to ensure sufficient power at all frequencies, including higher frequencies.

#### Experiment III.

The goal of the experiment was to examine whether integration windows reflected absolute time or the rate at which information varies in sound. Stimuli were similar to those for Experiment I except that we only tested speech and ferret vocalizations (not music), and we altered the information rate of the sounds by stretching and compressing the sounds (without altering the pitch). Sounds were was stretched and compressed by a factor of $\sqrt{2}$ and $\sqrt{\frac{1}{2}}$ prior to subdivision into segments using a phase vocoder with identity phase locking (Driedger, Müller, et al., 2014; Driedger, Muller, et al., 2014). Segments were excerpted from 16 natural sounds (500 ms, half speech, half ferret vocalizations) using the same range of segment durations as Experiment I. We also tested natural rate sounds without any stretching or compression.

### Data analysis

#### Selecting electrodes with a reliable response to sound.

We selected units (single and multi-units) with a reliable response to sound. Specifically, we measured the split-half Pearson correlation of the response timecourse of each unit across all stimuli from two non-overlapping sets of stimulus repetitions (odd vs. even). For Experiments I & II, we selected electrodes with a split-half correlation of at least 0.1, where this correlation was highly significant (p< 10^−5^). We used a lower absolute correlation threshold (0.05) in Experiment III because we were focusing on PEG which has sparser and more variable responses. We identified 693 units across 8 animals and 3 experiments that showed a reliable response to natural sounds based on these criteria (Exp I: 345, Exp II: 231, and Exp III: 117). Significance was measured using a permutation test where we permuted 100 ms segments of the neural timecourse (1000 permutations), and re-measured the correlation coefficient to build-up a null distribution. This null distribution was then fitted with a Gaussian from which we then computed the tail probability of the observed correlation value. We used a Gaussian fit rather than the empirical distribution so that we could measure small p values that would be impossible to measure via counting.

#### Cross-context correlation.

We measured integration windows using an analysis developed in prior work for estimating context invariance. The neural response timecourse to each sequence is organized as a segment x time matrix, where each row contains the response timecourse surrounding a single segment, aligned to segment onset (Figure 1B). A separate matrix is calculated for each of the two contexts, using the two different segment orderings/sequences. Corresponding rows contain the response timecourse to the same segment across the two different contexts. Different columns contain the responses from multiple segments for a fixed time lag relative to segment onset.

To estimate context invariance, we correlate corresponding columns (i.e. columns with the same lag) across the matrices from each of the two contexts (Figure 1B). If the integration window is less than the segment duration, there will be a moment when the response is the same across contexts yielding a correlation of 1. Because the correlation will never be 1 due to noise, we computed a noise ceiling for our measure by performing the same calculation when the context was identical using repeated presentations of each sequence.

#### Model-estimated windows.

In our prior work, we developed a computational model that is capable of using the cross-context correlation from multiple segment durations and lags to estimate a single underlying integration window (S. V. Norman-Haignere et al., 2022). We have extensively tested and validated the method in this prior work, showing that we can recover accurate integration window estimates from multiple ground truth models using noisy data.

The complete details of the method are described in our prior paper (S. V. Norman-Haignere et al., 2022). In brief, the integration window is parametrized with a Gamma distribution. We then make a prediction for the noise-free cross-context correlation by measuring the relative overlap of the window with shared central segments vs. surrounding context segments. This noise free prediction is then multiplied by the empirically measured noise ceiling to arrive at a prediction for the measured cross-context correlation. We determine the best fitting window by varying the window’s width and center, and selecting the window that yields the best prediction of the cross-context correlation. The width is defined as the central 75% of the window with the smallest mass and the center of the window is defined as the window’s median. We also varied the shape of the window, from more exponential to more Gaussian, although we have found that the shape does not substantially influence the predictions and results. The complete model includes several additional details that we have found improve the accuracy of the model in simulations. A detailed description and derivation of the added features is described in our prior paper.

#### Anatomical analysis - cortical surface maps.

There is variability in the functional location of cortical fields across animals. To create a cortical surface representation of all recorded units and to account for functional variability, we localized each electrode array on a standardized template tonotopic map, computed across many ferrets (J. K. Bizley & King, 2009; Nelken et al., 2004). The location of each array was selected based on the frequency selectivity of the electrode responses within that array, the tonotopic gradient found across the array and its position relative to anatomical landmarks. Electrode recordings from different sessions were assumed to sample different cells, and each recording was therefore plotted at the same location with a small amount of jitter so they could be distinguished. The jitter was sampled using polar coordinates (radius was sampled from a uniform distribution from 0 to 360° and distance was sampled from a uniform distribution from 20 to 240 micrometers). Recordings from the left hemisphere were mirrored onto the right hemisphere for visualization purposes. We measured the distance on the cortical surface of each unit to a reference point in the middle of primary auditory cortex.

#### Anatomical analysis - laminar assignment.

Laminar analysis was conducted following a method presented in our previous paper (López Espejo & David, 2024). In summary, cortical layers were categorized into three groups (layers 1–3, layer 4, and layers 5–6) and assigned to each electrode of the silicon probes using a custom graphical user interface. Boundaries between these groups were determined based on characteristic features of the local field potential (LFP) signal. The LFP signal was extracted by applying a zero-phase shift, 4th-order Butterworth low-pass filter (250 Hz, filter-filter method) to the raw electrophysiological data, followed by downsampling to 500 Hz. Key features used for layer identification included the current source density (CSD) sink and source patterns elicited by broadband noise bursts centered on each site’s best frequency (BF), which align with established auditory-evoked CSD patterns in the auditory cortex of other species (Schaefer et al., 2015). Additionally, the relative power of high-frequency (>40 Hz) versus low-frequency (<30 Hz) components of the LFP was analyzed to delineate layers 1–3, leveraging prior findings of increased high-frequency power in superficial cortical layers (Mendoza-Halliday et al., 2024). Finally, layer 4 was identified by detecting a drop in LFP coherence between adjacent channels at its upper boundary, consistent with earlier reports (Davis et al., 2023; Maier et al., 2010). Each unit was assigned to a cortical layer based on the electrode exhibiting the highest spike amplitude for that unit.

#### Rate-yoking index.

Compressing and stretching change the rate at which information varies by a known magnitude. We used a stretching and compression factor of $\sqrt{2}$ such that there is an octave difference in the rate at which information varies between stretched and compressed sounds. Thus, if the integration window reflects the information rate, we should observe a one-octave change in the integration window. We therefore computed a rate-yoking index by computing the difference in integration windows on an octave scale between stretched and compressed speech and dividing this difference by that which would be expected for a rate-yoked window (i.e., a one-octave change):

$$Rate-yoking\;index=\frac{\log_{2}\left(i_{stretched}\right)-\log_{2}\left(i_{compressed}\right)}{log_{2}\left(2\right)}$$

where i is the measured integration window for stretched/compressed sounds. An index of 0 indicates a fixed, time-yoked window, while a value of 1 indicates a fully rate-yoked window. Note that the denominator in the above equation happens to be 1, and thus has no effect on the result, since there was an octave difference in the information rate for stretched and compressed sounds. If we had used a different stretching and compression factor, then it would be different and would need to be included. The rate-yoking index is not strictly bound between 0 and 1 and noise will tend to cause the estimate to exceed these bounds. A value less than 0 indicates that the integration window was longer for compressed sounds and a value greater than 1 indicates that the difference between stretched and compressed integration windows exceeded 1 octave.

#### Statistics.

Unless otherwise noted, we used a linear mixed effects (LME) model to evaluate the significance of our effects (fitlme.m and coefTest.m in MATLAB; Satterthwaite approximation was used to estimate degrees of freedom when measuring significance). Whenever possible, we included random effects to account for variation across sessions from day to day, using diagonal covariance matrices to prevent overfitting/singular results. As is typical, we do not include random effects for animals because of the small number of animals in our experiment. For categorical variables, the category with the most samples was always used as the baseline and not modeled explicitly in the linear model. Integration window estimates were always logarithmically transformed before being fit by the LME model.

For Experiment I, to test whether integration windows increased across the cortical hierarchy, we modeled the integration window as a linear function of distance to primary auditory cortex, including random intercepts and slopes for different recording sessions:

$$y∼1+distance+\left(1+distance\;|\;session\right)$$

where y reflects either the integration width or center (logarithmically transformed). The sample dimension was unit (single or multi-unit).

To test whether integration windows varied across regions, we used the following model:

$$y∼1+region+\left(1+region\;|\;session\right)$$

Region was a binary categorical variable (MEG vs. PEG).

For Experiment II, which used laminar probes, we investigated effects of layer and region using the following model:

$$y∼1+layer+region+\left(1\;|\;session\right)$$

Layer was a categorical variable with three levels (granular, infragranular, supragranular). Region was again a binary variable (MEG, PEG). A different insertion was used for each recording session, and as a consequence, session was partially confounded with region and layer and we found that a model with random slopes did not converge (and these terms were thus removed). We tested whether the effect of region was significantly different (larger) than the effect of layer by directly comparing their coefficients against a null model that assumed no difference. To make this a fair comparison, we collapsed our layers from three levels to two levels to make them comparable to that for each region. To be conservative, we chose the two levels so that the effect of layers would be maximal thus minimizing the likelihood that the effect of the region would be even larger than the effect of layer. To this end, we separated out the infragranular layers from the granular and supragranular layers (infragranular vs. granular/supragranular), because the infragranular layers showed the most different integration windows (Figure 3).

For Experiment III, we used the following model to investigate the effect of rate, controlling for any possible difference due to sound category (ferrets vs. speech):

$$y∼1+rate+category+\left(1\;\text{|}\;unit\right)+\left(1\;\text{|}\;session\right)$$

Rate and category were categorical variables with three (original, compressed, speech) and two (speech, ferrets) levels, respectively. We did not include random slopes for session in this model because the model was not able to estimate them (variance terms for these random effects were either undefined or close to 0).

## Supplementary Material

Supplement 1

## Data Availability

Code and data necessary for replication of the results in this article will be made publicly available upon publication in a peer reviewed journal.
