# Supplementary material for: Neurons in auditory cortex integrate information within constrained temporal windows that are invariant to the stimulus context and information rate
Source: bioRxiv. 2025 Sep 16:2025.02.14.637944. Originally published 2025 Feb 14. Preprint. [Version 2] doi: 10.1101/2025.02.14.637944 (PMC11844508; doi:10.1101/2025.02.14.637944)
Supplement: 1 [file NIHPP2025.02.14.637944V2-supplement-1.pdf]

bioRxiv preprint doi: https://doi.org/10.1101/2025.02.14.637944; this version posted September 16, 2025. The copyright holder for this preprint (which was not certified by peer review) is the author/funder, who has granted bioRxiv a license to display the preprint in perpetuity. It is made available under aCC-BY 4.0 International license.

Supplementary Figures

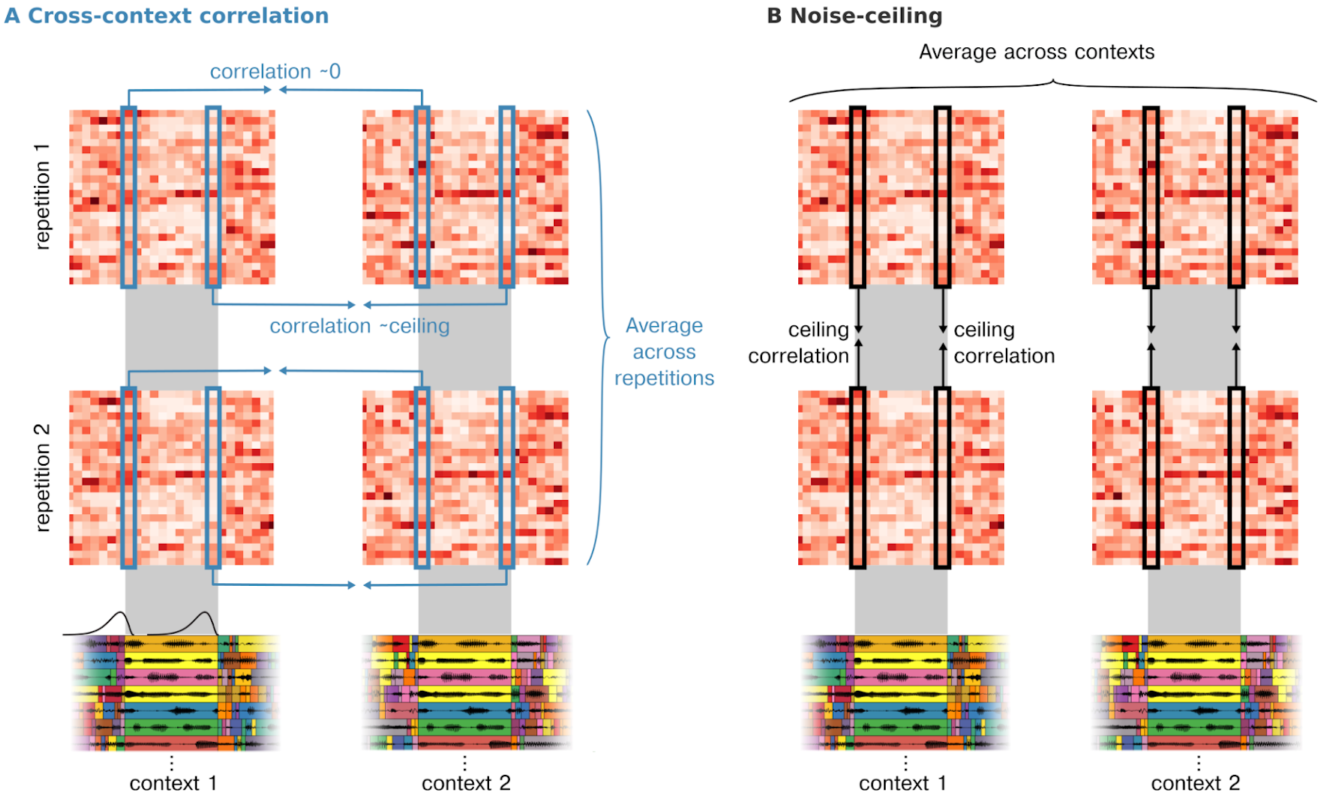

Supplementary Figure 1 | More detailed schematic of the cross-context correlation (**A**) and noise-ceiling (**B**) as applied to an example unit. This legend gives a brief description of the core ideas as they are linked to this figure. See the text for a more in-depth description of the analyses. **A**, The neural response timecourse of the unit is reorganised as a matrix. Specifically, the response timecourse surrounding all of the segments of a given duration (500 ms in this schematic) was compiled into a segment-by-time matrix, aligned to segment onset (as in **Figure 1B**). The gray shaded area shows the time period when the shared segment was present. Each row contains the response timecourse to a single segment, and each column contains the response to many segments for a single time lag relative to segment onset. Separate matrices were computed for two different contexts (context 1: left, context 2: right) and two different repetitions of the same context (rep 1: top, rep 2: bottom). Below the matrices, we plot waveforms of corresponding segments: the top waveform corresponds to the segment for the first row of the matrix, the second waveform from the top corresponds to the segment of the second row, and so on. The cross-context correlation is computed by correlating corresponding columns of matrices from different contexts (blue columnar boxes), separately for each repetition, and then averaging the correlation coefficients across repetitions. The correlation is computed separately for each time lag relative to segment onset, and a schematic of the hypothesized integration window at each time lag is shown below, overlaid on the stimulus waveforms. At segment onset, the integration window will fall on the preceding context segments, which are independent across contexts, and the correlation should thus be approximately 0. If the integration window is less than the segment duration, then there will be a moment when the window is fully contained within the shared segment, and at this moment, the cross-context correlation should equal the maximum possible value given by the noise ceiling. One can visually observe that the responses from this unit become more similar as time progresses within the segment. To make it possible to visually observe the key trends in this figure, we used a 50 ms bin. Quantitative analyses were performed using a much smaller bin (5 ms) to ensure that the bin size did not upward-bias the measured integration window. **B**, To compute the noise ceiling, we correlated columns across repetitions, separately for each context, and averaged the correlation coefficient across contexts. Because the context is

bioRxiv preprint doi: https://doi.org/10.1101/2025.02.14.637944; this version posted September 16, 2025. The copyright holder for this preprint (which was not certified by peer review) is the author/funder, who has granted bioRxiv a license to display the preprint in perpetuity. It is made available under aCC-BY 4.0 International license.

identical, the noise ceiling provides an upper bound of the maximum possible correlation that could be observed when comparing responses across different contexts.

bioRxiv preprint doi: https://doi.org/10.1101/2025.02.14.637944; this version posted September 16, 2025. The copyright holder for this preprint (which was not certified by peer review) is the author/funder, who has granted bioRxiv a license to display the preprint in perpetuity. It is made available under aCC-BY 4.0 International license.

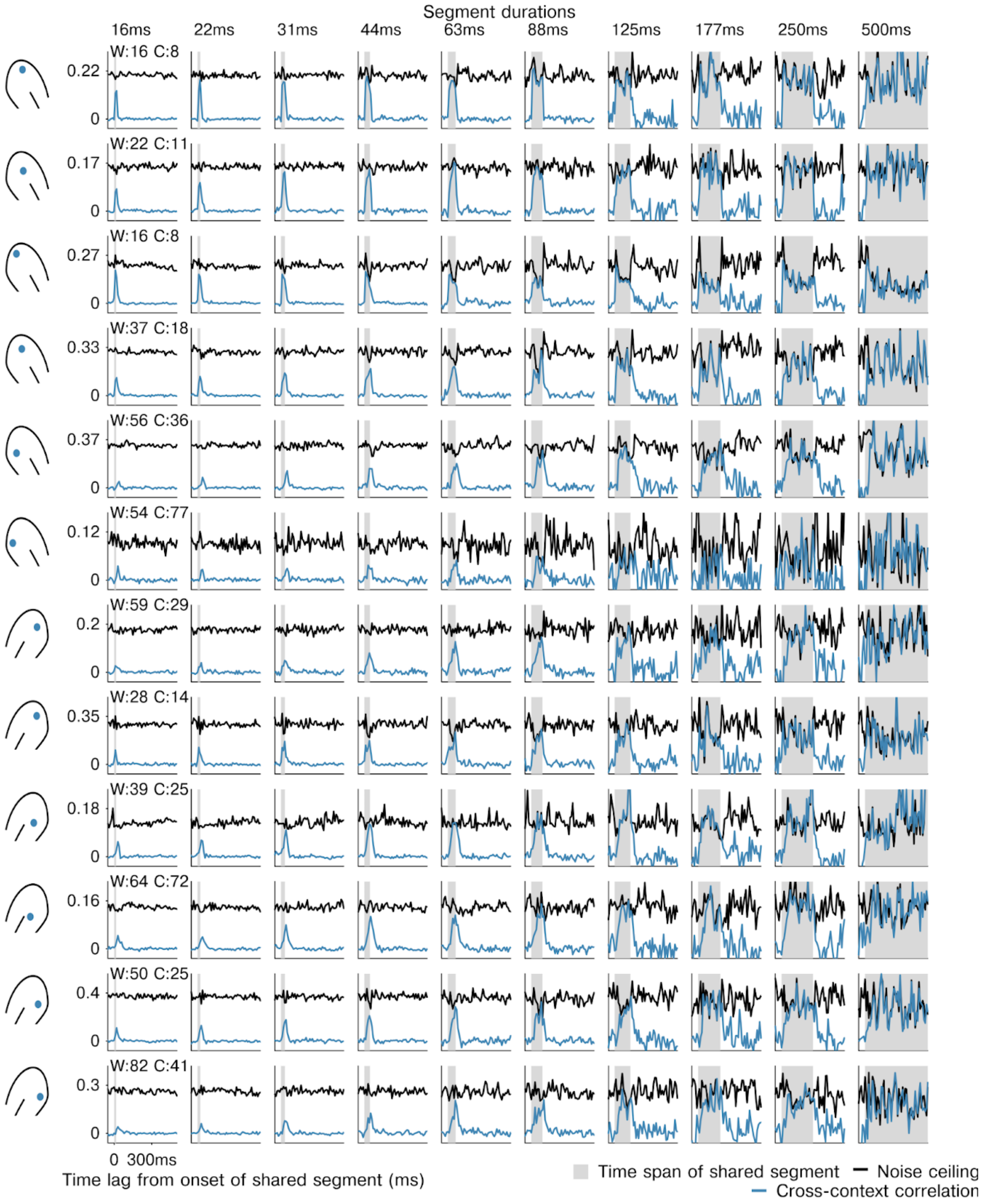

Supplementary Figure 2 | Examples of cross-context correlation for multi-units in different regions of the auditory cortex. Examples of the CCC (blue) and noise ceiling (black) from example multi-units from primary and

bioRxiv preprint doi: https://doi.org/10.1101/2025.02.14.637944; this version posted September 16, 2025. The copyright holder for this preprint (which was not certified by peer review) is the author/funder, who has granted bioRxiv a license to display the preprint in perpetuity. It is made available under aCC-BY 4.0 International license.

non-primary ferret auditory cortex. For all units, there is a lag and segment duration for which CCC equals the noise ceiling, indicating a context-invariant response. The segment duration needed to achieve a context-invariant response varies substantially across units. The location of each recorded multi-unit is indicated to the left of each row.

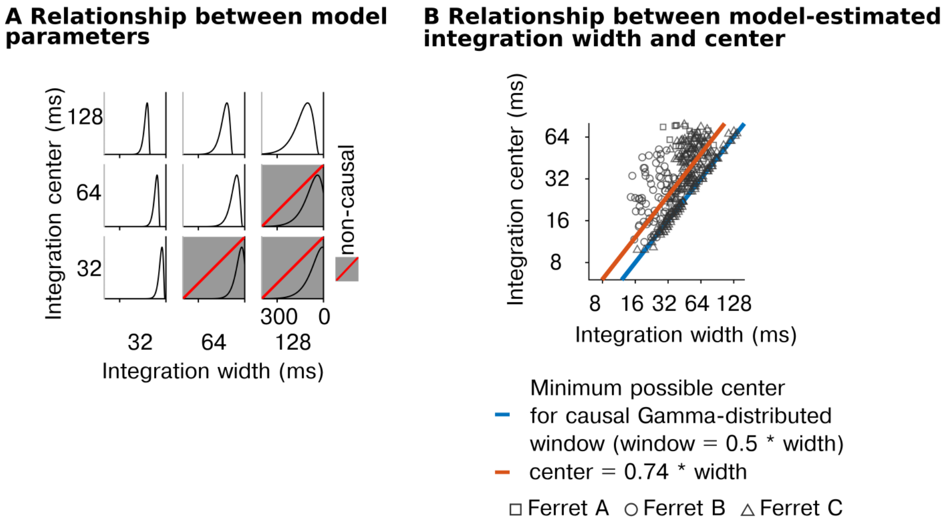

Supplementary Figure 3 | Relationship between the center and width of the integration window. A, Integration windows were estimated using a parametric window (Gamma distribution) with a varying width and center. We estimated the width and center that best predicted the cross-context correlation, excluding parameters that yielded a non-causal window. This panel plots examples of the parameter window as a function of the window's width (x-axis) and center (y-axis). Combinations of parameters that led to acausal windows were excluded (gray box with red dashed line) because they are not biologically possible. **B**, Scatter plot of estimated integration centers vs widths for all units with a best-fit linear function overlaid (orange line). The blue line shows the minimum possible center for a causal, Gamma-distributed window.

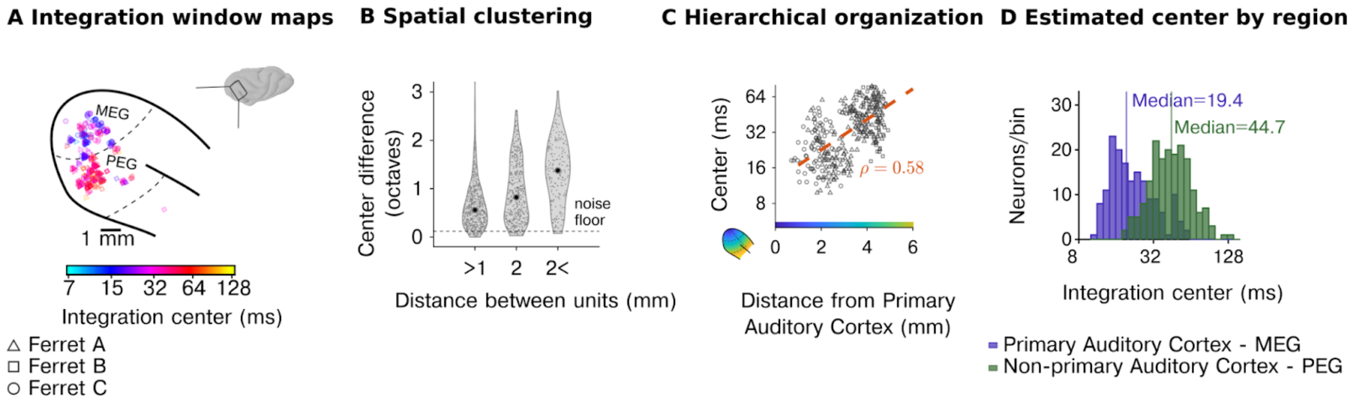

Supplementary Figure 4 | Organisation of integration centers across neural populations in the auditory cortex. A, Anatomical map of model-estimated integration centers in three animals (window center: median of the interval containing 75% of the window's mass). **B**, The difference between the centers of the integration windows between pairs of units as a function of their spatial distance, demonstrating that nearby units have more similar centers. **C**, Centers of the integration windows as a function of distance to the primary auditory cortex (see color

bioRxiv preprint doi: https://doi.org/10.1101/2025.02.14.637944; this version posted September 16, 2025. The copyright holder for this preprint (which was not certified by peer review) is the author/funder, who has granted bioRxiv a license to display the preprint in perpetuity. It is made available under aCC-BY 4.0 International license.

map in inset). **D**, Histograms of the centers of the integration windows for primary and non-primary auditory cortex showing substantial diversity across units and hierarchical organization.

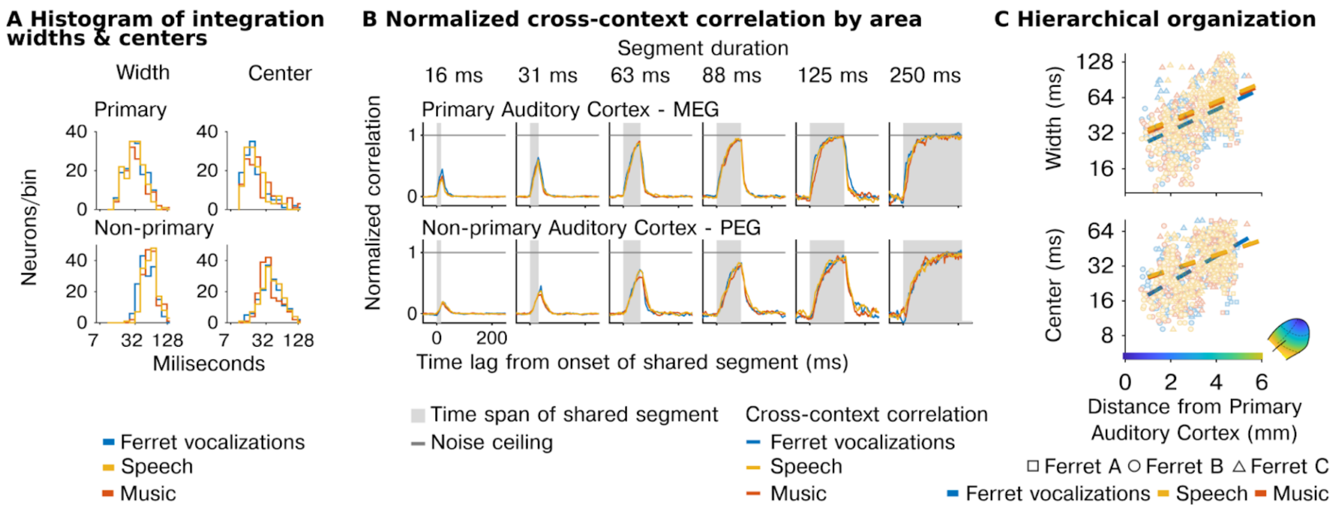

Supplementary Figure 5 | Temporal integration is similar for different stimulus categories. A, Histograms of model-fitted integration window parameters (widths and centers) for units in primary and non-primary auditory cortex across the three sound categories tested (ferret vocalizations, speech, music). **B**, Normalized median cross-context correlation for primary and non-primary cortex, plotted separately for each sound category. **C**, Scatter plots of the model-fitted integration centers and widths as a function of distance to primary auditory cortex computed separately for each category with best-fit lines.

bioRxiv preprint doi: https://doi.org/10.1101/2025.02.14.637944; this version posted September 16, 2025. The copyright holder for this preprint (which was not certified by peer review) is the author/funder, who has granted bioRxiv a license to display the preprint in perpetuity. It is made available under aCC-BY 4.0 International license.

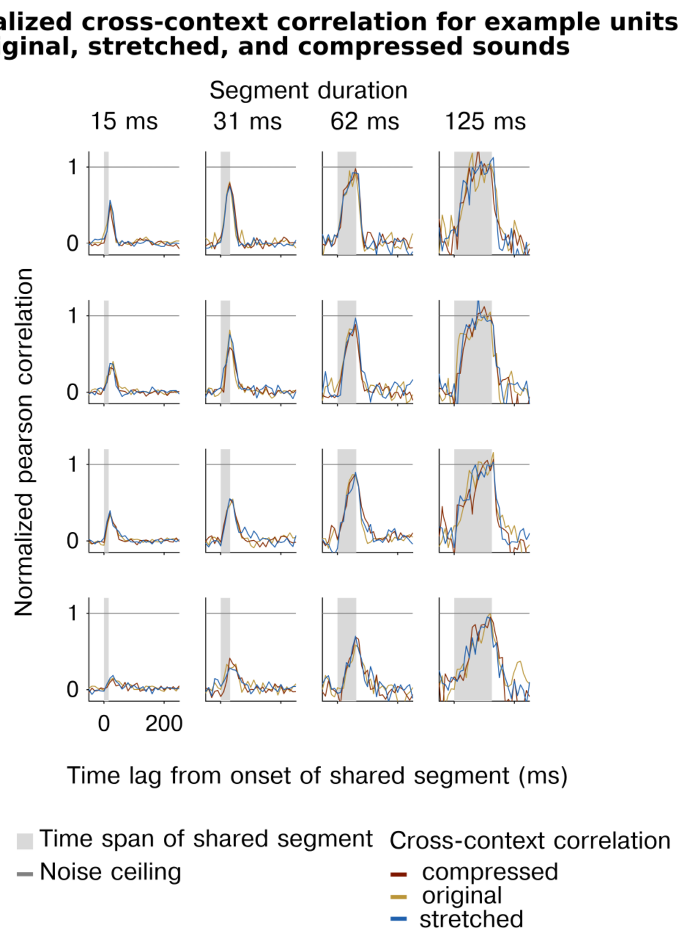

Supplementary Figure 6 | Normalized cross-context correlation for example multi-units for different sound rates. Normalized cross-context correlation for five individual units (rows) plotted separately for each of the three different sound rates (compressed, original, stretched). The normalized CCC was computed by dividing the CCC by the noise ceiling separately for every segment duration and time point.

ly.

bioRxiv preprint doi: https://doi.org/10.1101/2025.02.14.637944; this version posted September 16, 2025. The copyright holder for this preprint (which was not certified by peer review) is the author/funder, who has granted bioRxiv a license to display the preprint in perpetuity. It is made available under aCC-BY 4.0 International license.

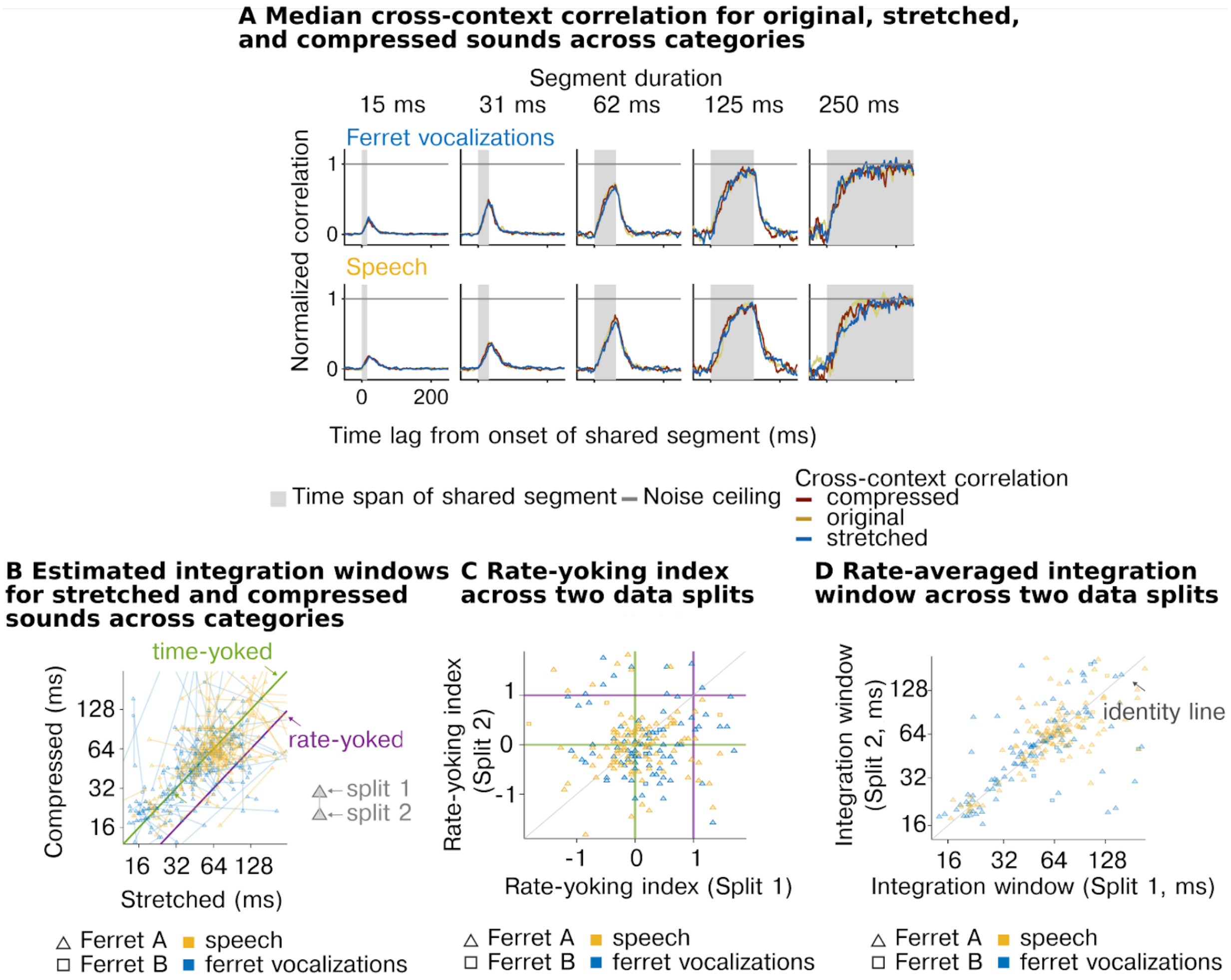

Supplementary Figure 7 | Time-yoked integration is consistent across sound categories. Format is the same as Figure 4, but plotting results separately for speech and ferret vocalisations. **A**, Median normalized CCC across all recorded units for stretched, original, and compressed stimuli computed separately for both sound categories tested (ferret vocalizations and speech). **B**, Integration windows (widths) of all units for compressed (x-axis) and stretched (y-axis) stimuli plotted separately for each sound category (speech in yellow and ferret vocalizations in blue). Green and purple lines show the prediction from a time-yoked vs. rate-yoked response. **C**, Reliability of rate-yoking index across two independent data splits, separately for each category. **D**, Reliability of integration windows averaged across stimuli rates for comparison, again separately for each category.

bioRxiv preprint doi: https://doi.org/10.1101/2025.02.14.637944; this version posted September 16, 2025. The copyright holder for this preprint (which was not certified by peer review) is the author/funder, who has granted bioRxiv a license to display the preprint in perpetuity. It is made available under aCC-BY 4.0 International license.

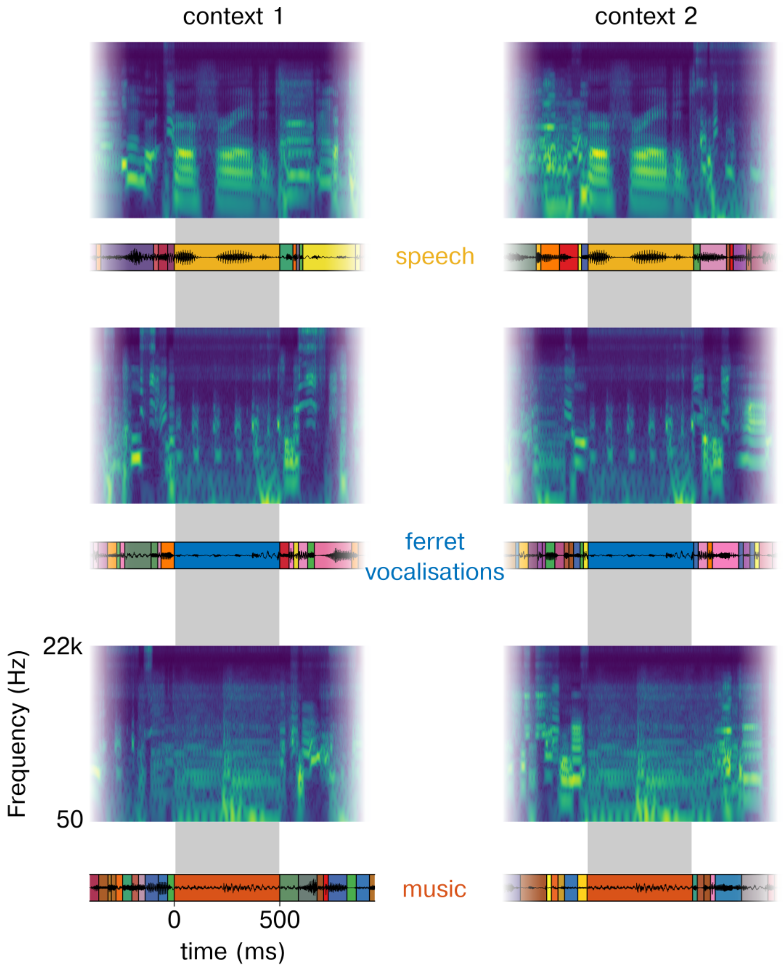

Supplementary Figure 8 | Example spectrograms of the segment sequences used in Experiment I. Each row shows a different example segment (top: speech segment, middle: ferret vocalisation segment: bottom: music segment). The left and right panels show spectrograms of the same segment in two different contexts.

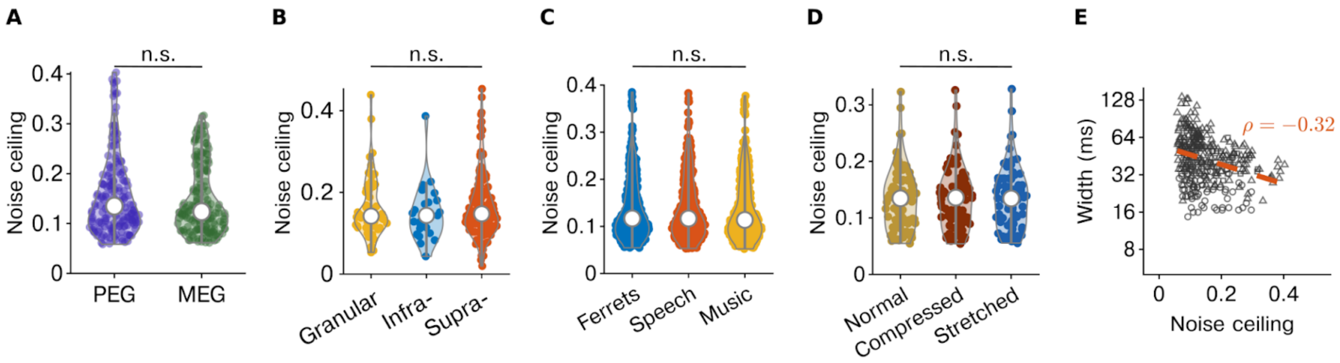

Supplementary Figure 9 | Noise ceiling across conditions and regions. A, Histograms of the distribution of the noise-ceiling in the Primary (PEG) and Non-Primary (MEG) auditory cortex. **B**, Violin plot of the average

bioRxiv preprint doi: https://doi.org/10.1101/2025.02.14.637944; this version posted September 16, 2025. The copyright holder for this preprint (which was not certified by peer review) is the author/funder, who has granted bioRxiv a license to display the preprint in perpetuity. It is made available under aCC-BY 4.0 International license.

noise-ceiling for all units recorded in Experiment I across the sound categories. **C**, Violin plot of the average noise-ceiling for all units recorded in Experiment II across the layers. **D**, Violin plot of the average noise-ceiling for all units recorded in Experiment III for the 3 experimental conditions. **E,** Scatter plot representing the relationship between the noise ceiling and integration width (left) and center (right). Orange lines show affine fits, with Spearman correlation coefficients indicated.

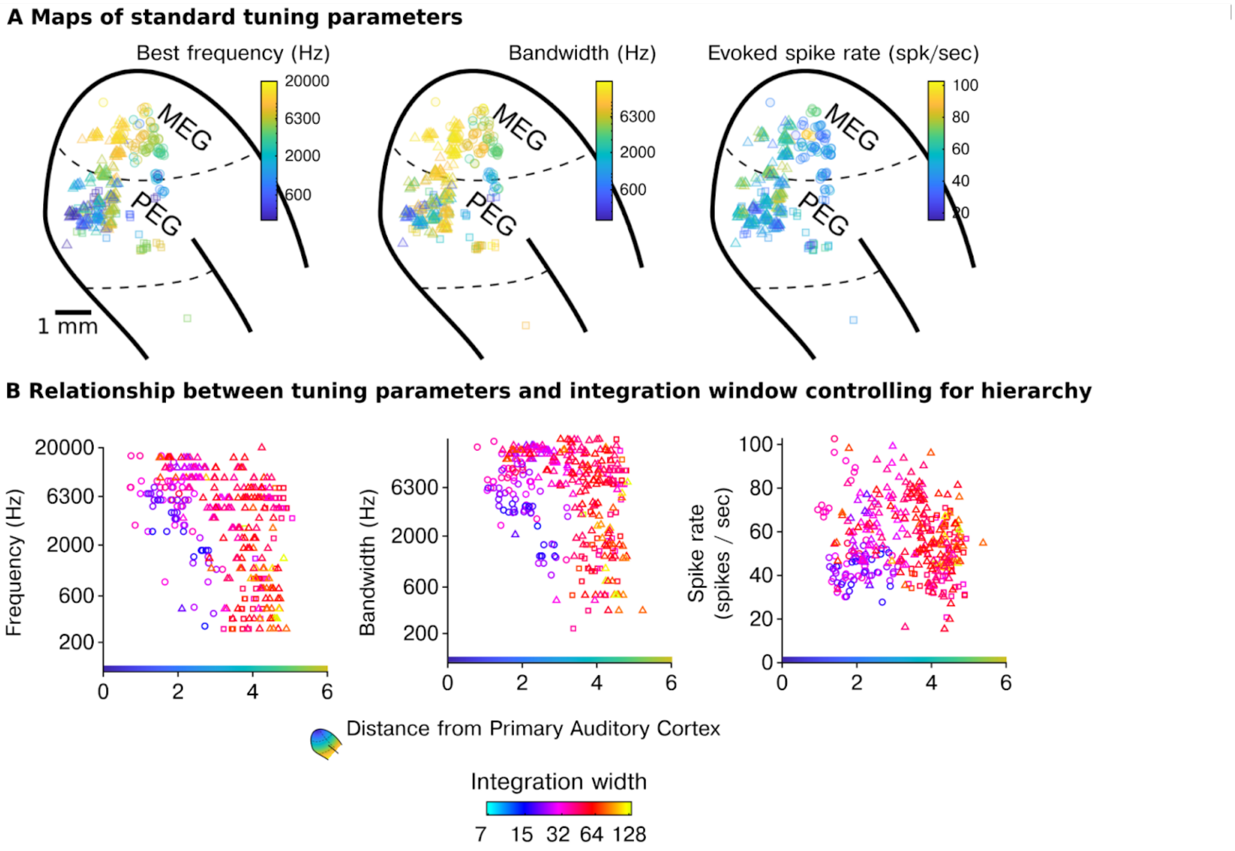

Supplementary Figure 10 | Relationship between standard tuning parameters and neural integration window. A, Maps of standard tuning parameters. **B,** Scatter plots show the tuning parameters for each cell plotted against a measure of anatomical hierarchy (distance to primary auditory cortex) with the integration window indicated by color.
