# Supplementary material for: Neurons in auditory cortex integrate information within constrained temporal windows that are invariant to the stimulus context and information rate
Source: bioRxiv. 2025 Feb 14:2025.02.14.637944. Preprint. [Version 1] doi: 10.1101/2025.02.14.637944 (PMC11844508; doi:10.1101/2025.02.14.637944)
Supplement: Supplementary file 1 [file NIHPP2025.02.14.637944v1-supplement-1.pdf]

bioRxiv preprint doi: https://doi.org/10.1101/2025.02.14.637944; this version posted February 14, 2025. The copyright holder for this preprint (which was not certified by peer review) is the author/funder, who has granted bioRxiv a license to display the preprint in perpetuity. It is made available under aCC-BY 4.0 International license.

Supplementary Figures

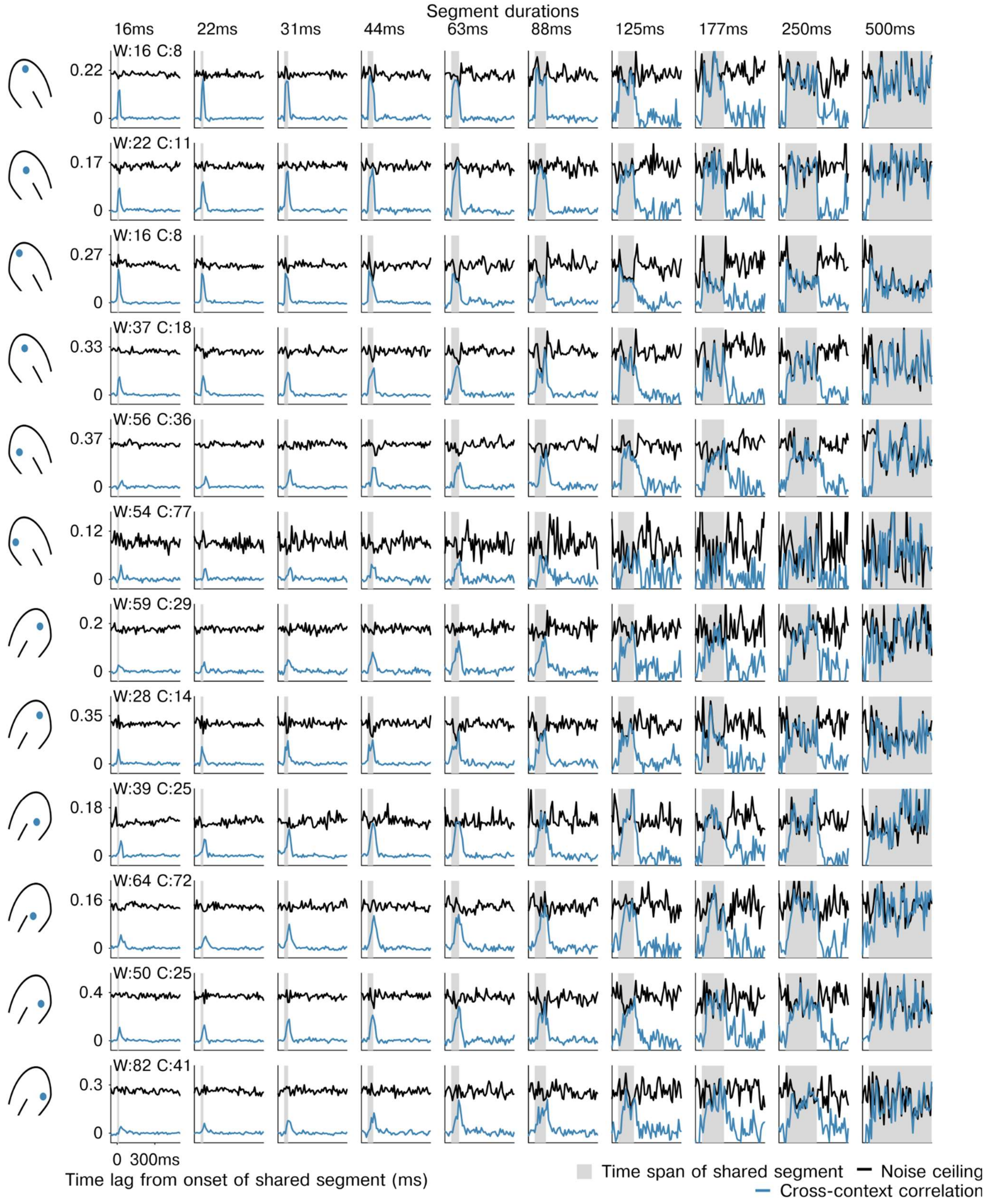

Supplementary Figure 1 | Examples of cross-context correlation for multi-units in different regions of the auditory cortex. Examples of the CCC (blue) and noise ceiling (black) from example

bioRxiv preprint doi: https://doi.org/10.1101/2025.02.14.637944; this version posted February 14, 2025. The copyright holder for this preprint (which was not certified by peer review) is the author/funder, who has granted bioRxiv a license to display the preprint in perpetuity. It is made available under aCC-BY 4.0 International license.

multi-units from primary and non-primary ferret auditory cortex. For all units, there is a lag and segment duration for which CCC equals the noise ceiling, indicating a context-invariant response. The segment duration needed to achieve a context-invariant response varies substantially across units. The location of each recorded multi-unit is indicated to the left of each row.

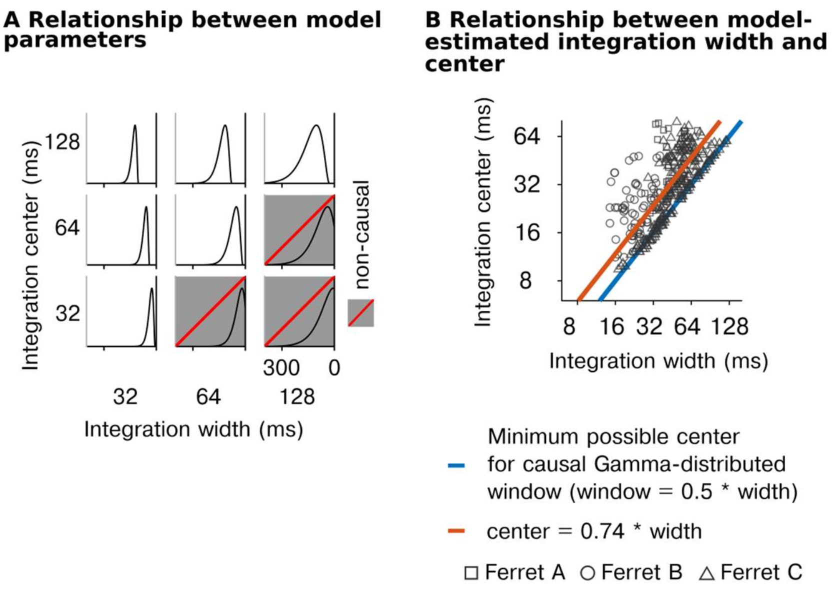

Supplementary Figure 2 | Relationship between the center and width of the integration window. A, Integration windows were estimated using a parametric window (Gamma distribution) with a varying width and center. We estimated the width and center that best predicted the cross-context correlation, excluding parameters that yielded a non-causal window. This panel plots examples of the parameter window as a function of the window's width (x-axis) and center (y-axis). Combination of parameters that led to acausal windows were excluded (gray box with red dashed line) because they are not biologically possible. **B**, Scatter plot of estimated integration centers vs widths for all units with a best-fit linear function overlaid (orange line). Blue line shows the minimum possible center for a causal, Gamma-distributed window.

bioRxiv preprint doi: https://doi.org/10.1101/2025.02.14.637944; this version posted February 14, 2025. The copyright holder for this preprint (which was not certified by peer review) is the author/funder, who has granted bioRxiv a license to display the preprint in perpetuity. It is made available under aCC-BY 4.0 International license.

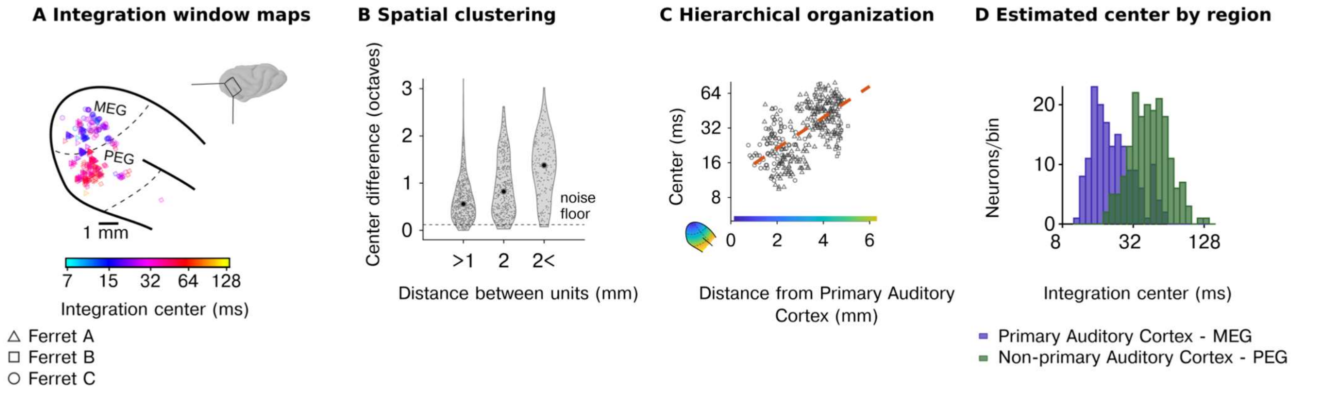

Supplementary Figure 3 | Organisation of integration centers across neural populations in the auditory cortex. A, Anatomical map of model-estimated integration centers in three animals (window center: median of the interval containing 75% of the window's mass). **B**, The difference between the centers of the integration windows between pairs of units as a function of their spatial distance, demonstrating that nearby units have more similar centers. **C**, Centers of the integration windows as a function of distance to the primary auditory cortex (PAC) (see color map in inset). **D**, Histograms of the centers of the integration windows for primary (MEG) and non-primary auditory cortex (PEG) showing substantial diversity across units and hierarchical organization.

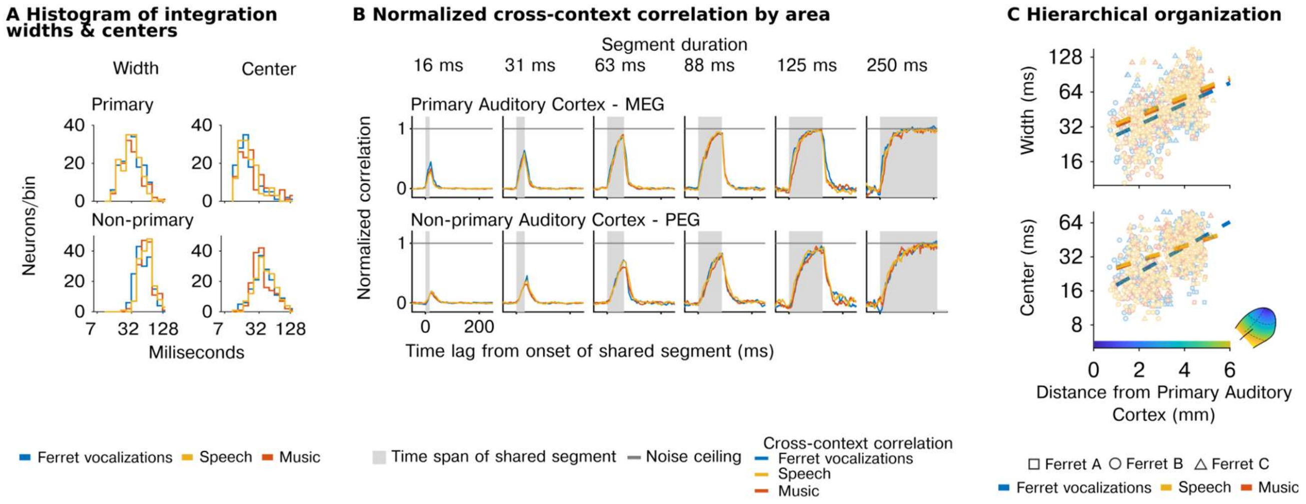

Supplementary Figure 4 | Temporal integration is similar for different stimulus categories. A, Histograms of model-fitted widths and centers of neurons in primary and non-primary auditory cortex across the three sound categories tested (Ferret vocalizations, Speech, Music). **B**, Normalized median cross-context correlation split by sound category across the two cortical areas. **C**, Scatter plots of the model-fitted integration centers and widths as a function of distance to primary auditory cortex for all three sound categories tested.

bioRxiv preprint doi: https://doi.org/10.1101/2025.02.14.637944; this version posted February 14, 2025. The copyright holder for this preprint (which was not certified by peer review) is the author/funder, who has granted bioRxiv a license to display the preprint in perpetuity. It is made available under aCC-BY 4.0 International license.

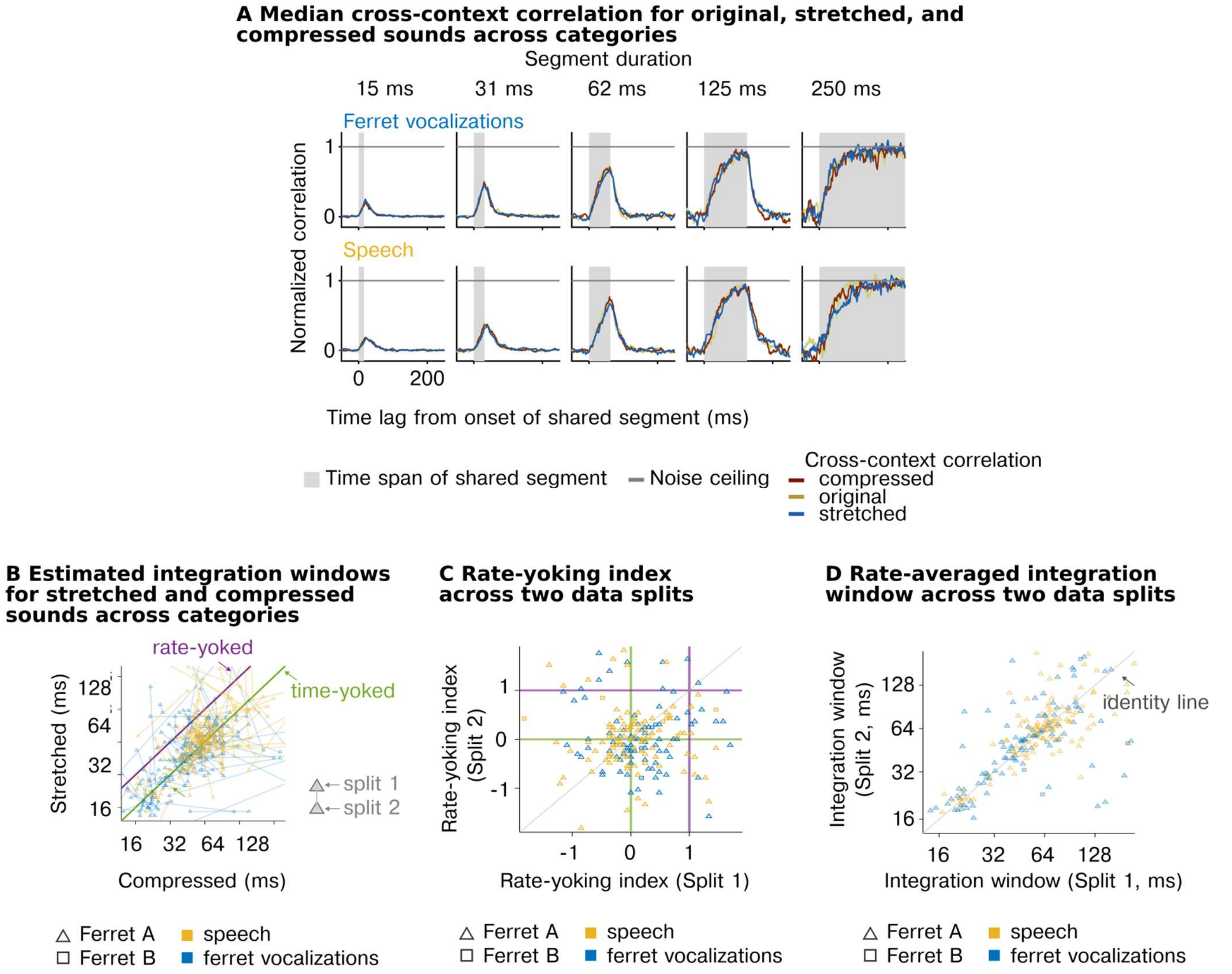

Supplementary Figure 5 | Time-yoked integration is consistent across sound categories. A, Median normalized CCC across all recorded units for stretched, original, and compressed stimuli for both sound categories (Ferret vocalizations and Speech). **B**, Integration windows of all units for compressed (x-axis) and stretched (y-axis) stimuli for both sound categories, separately (speech in yellow and ferret vocalizations in blue). Green and purple lines show the prediction from a time-yoked and rate-yoked response. **C**, Reliability of rate-yoking index across two independent data splits, separately for each category. **D**, Reliability of integration windows averaged across stimuli rates for comparison, again separately for each category.
